# Supplementary material for: Transcriptomic profiling of lung alveolar macrophages reveals distinct contribution of sterol metabolism in macrophage response to Cryptococcus gattii infection
Source: PLoS One. 2025 Sep 30;20(9):e0333090. doi: 10.1371/journal.pone.0333090 (PMC12483273; doi:10.1371/journal.pone.0333090)
Supplement: S1 Table — (DOCX) [file pone.0333090.s001.docx]

**Table S1. Data quality summary of purified alveolar macrophage samples from PBS-treated, *C. gattii*-infected and *C. neoformans*-infected mice at 7 days after infection.**

| Sample Name | Raw Reads | Clean Reads | Raw Bases | Clean Bases | Error Rate (%) | Q20 (%) | Q30 (%) | GC Content (%) |
| --- | --- | --- | --- | --- | --- | --- | --- | --- |
| PBS | 39,172,080 | 34,512,034 | 5.9G | 5.2G | 0.03 | 93.41 | 86.52 | 50.35 |
| Cg_D_7 | 39,351,234 | 35,312,258 | 5.9G | 5.3G | 0.02 | 94.65 | 88.14 | 49.67 |
| Cn_D_7 | 43,236,486 | 41,618,054 | 6.5G | 6.2G | 0.02 | 96.70 | 91.82 | 50.96 |

Q20, PhredQscore20; Q30, PhredQscore30
