# Supplementary material for: Transcriptomic profiling of lung alveolar macrophages reveals distinct contribution of sterol metabolism in macrophage response to Cryptococcus gattii infection
Source: PLoS One. 2025 Sep 30;20(9):e0333090. doi: 10.1371/journal.pone.0333090 (PMC12483273; doi:10.1371/journal.pone.0333090)
Supplement: S2 Table — (DOCX) [file pone.0333090.s002.docx]

**Table S2. Primers used for quantitative real-time PCR analysis.**

| Gene | Primer sequence (5’-3’) |
| --- | --- |
| Mouse *Fdps* | Forward: GCG TTG AAG AAC AGA GCA TTT AG  Reverse: CGC AAT AGG CAG GTA GAA AGA |
| Mouse *Cyp51* | Forward: CGA GGA TCT GCC TCC TTT AAC  Reverse: GGC CTC AGT CTT AGT GTT TCT T |
| Mouse *Sqle* | Forward: GGA GTC CAA GAA GTG CAG AAA  Reverse: CAG ATC CAA GCA CAC CAG AT |
| Mouse *Sc5d* | Forward: GTC TTT GCC TTG GAT CAG CAT CC  Reverse: GGA AGG ATA CGA CGC TAA CCA TG |
| Mouse *Hsd17b7* | Forward: CTG TGA CAC CGT ACA ACG GA  Reverse: GCT CGG GTG ATC CGA TTT CT |
| Mouse *Fdft1* | Forward: CAA ACA GGA CTG GGA CAA GTA  Reverse: GAG GCA GAG ATT AGA CGA GAA AG |
| Mouse *Msmo1* | Forward: CTG TGC AGT CAT TGA GGA CAC C  Reverse: GGG TTT CCA AGG GAT GTG CGT A |
| Mouse *Lss* | Forward: GCA CAC CAC AGA CCT GAG TTT C  Reverse: CAG TGT GCT GAA GGA GAA ACC AC |
| Mouse *Abca1* | Forward: AGG GTT TCT TTG CTC AGA TTG TC  Reverse: TGC CAA AGG GTG GCA CA |
| Mouse *Pltp* | Forward: GCT GCT GAA CAT CTC CAA CGC A  Reverse: GCT GTA GAC CTG TTC GGA TGG A |
| Mouse *Plin1* | Forward: TGA AGG GTG TTA CGG ATA ACG  Reverse: ATG TCT CGG AAT TCG CTC TC |
| Mouse *Cidec* | Forward: TCG GAA GGT TCG CAA AGG CAT C  Reverse: CTC CAC GAT TGT GCC ATC TTC C |
| Mouse *Nos2* | Forward: GGA GTG ACG GCA AAC ATG ACT  Reverse: TCG ATG CAC AAC TGG GTG AAC |
| Mouse *Il6* | Forward: TCT ATA CCA CTT CAC AAG TCG GA  Reverse: GAA TTG CCA TTG CAC AAC TCT TT |
| Mouse *Il1b* | Forward: CTG AAC TCA ACT GTG AAA T  Reverse: AAA GGT TTG GAA GCA GCC C |
| Mouse *Tnfa* | Forward: CAG GCG GTG CCT ATG TCT C  Reverse: CGA TCA CCC CGA AGT TCA GTA G |
| Mouse *Mmp9* | Forward: TGT ACA CAG GCA AGA CCG T  Reverse: CTC ATG GTC CAC CTT GTT C |
| Mouse *Arg1* | Forward: CTG CAT GGG CAA CCT GTG T  Reverse: CTT GGT ACA TCT GGG AAC TTT CC |
| Mouse *Fizz1* | Forward: TAC TTG CAA CTG CCT GTG CTT ACT  Reverse: TAT CAA AGC TGG GTT CTC CAC CTC |
| Mouse *Ym1* | Forward: TCT CTA CTC CTC AGA ACC GTC AGA  Reverse: GAT GTT TGT CCT TAG GAG GGC TTC |
| Mouse *Tlr4* | Forward: TGT CAT CAG GGA CTT TGC TG  Reverse: TGT TCT TCT CCT GCC TGA CA |
| Mouse *Lyz1* | Forward: GCC AAG GTA TAC AAT CGT TGT GAG TTG  Reverse: CAG TCA GCC AGC TTG ACA CCA CG |
| Mouse *Actb* | Forward: GAC GGC CAG GTC ATC ACT ATT G  Reverse: AGG AAG GCT GGA AAA GAG CC |

*Fdps*, Farnesyl Diphosphate Synthase; *Cyp51*, Cytochrome P450, Family 51; *Sqle*, Squalene Epoxidase; *Sc5d*, Sterol-C5-Desaturase; *Hsd17b7*, Hydroxysteroid 17-Beta Dehydrogenase 7; *Fdft1*, Farnesyl-Diphosphate Farnesyltransferase 1; *Msmo1*, Methylsterol Monooxygenase 1; *Lss*, Lanosterol Synthase; *Abca1*, ATP Binding Cassette Subfamily A Member 1; *Pltp*, Phospholipid Transfer Protein; *Plin1*, Perilipin 1; *Cidec*, Cell Death Inducing DFFA Like Effector C; *Nos2*, Nitric Oxide Synthase 2; *Il6*, Interleukin 6; *Il1b*, Interleukin 1 Beta; *Tnfa*, Tumor Necrosis Factor-Alpha; *Mmp9*, Matrix Metallopeptidase 9; *Arg1*, Arginase 1; *Fizz1*, Found Inflammatory Zone 1or Resistin Like Alpha; *Ym1*,  Chitinase 3-Like 3; *Tlr4*, Toll Like Receptor 4; *Lyz1*, Lysozyme 1; *Actb*, β-actin
