# Supplementary material for: Transcriptomic profiling of lung alveolar macrophages reveals distinct contribution of sterol metabolism in macrophage response to Cryptococcus gattii infection
Source: PLoS One. 2025 Sep 30;20(9):e0333090. doi: 10.1371/journal.pone.0333090 (PMC12483273; doi:10.1371/journal.pone.0333090)
Supplement: S3 Table — (DOCX) [file pone.0333090.s003.docx]

| Upregulated | | | Downregulated | | |
| --- | --- | --- | --- | --- | --- |
| Gene | Log_2_ Fold Change | P-value | Gene | Log_2_ Fold Change | P-value |
| *Iigp1* | 14.844 | 1.17E-50 | *Cd55* | -7.6805 | 3.61E-06 |
| *Tlr12* | 11.184 | 2.50E-24 | *Hbb-bs* | -6.0364 | 3.64E-29 |
| *Gbp11* | 11.112 | 7.45E-24 | *Hba-a2* | -5.7032 | 1.20E-17 |
| *Ocstamp* | 10.48 | 8.65E-20 | *Hba-a1* | -5.6806 | 4.81E-27 |
| *Gm4951* | 10.305 | 1.03E-39 | *Alas2* | -4.8487 | 1.53E-10 |
| *Tgtp1* | 10.108 | 1.68E-17 | *Impg2* | -4.8059 | 7.53E-06 |
| *Tmeff1* | 10.047 | 4.03E-17 | *Cyp4f37* | -4.6994 | 1.58E-05 |
| *Gm5970* | 9.9404 | 1.59E-16 | *Pappa* | -4.5234 | 4.96E-05 |
| *Tgtp2* | 9.7744 | 1.65E-40 | *F630028O10Rik* | -4.3692 | 8.61E-07 |
| *Ly6i* | 9.6327 | 9.32E-15 | *Bpifa1* | -4.3318 | 1.16E-06 |
| *Nos2* | 9.42 | 6.64E-38 | *Fabp1* | -4.2339 | 1.05E-18 |
| *C1qa* | 9.3451 | 2.65E-13 | *Arhgap22* | -4.0168 | 5.50E-10 |
| *Ddx4* | 9.2228 | 1.22E-12 | *Gpr34* | -3.9668 | 9.37E-10 |
| *Apol9a* | 9.1286 | 3.82E-12 | *Nlrp10* | -3.8928 | 4.06E-05 |
| *Ly6a* | 9.0279 | 1.27E-11 | *Pnpla5* | -3.6831 | 1.27E-06 |
| *Sectm1a* | 9.0069 | 1.27E-11 | *Prr33* | -3.674 | 6.76E-11 |
| *Serpina3f* | 8.8455 | 3.39E-29 | *Coro6* | -3.6099 | 4.54E-15 |
| *Btnl4* | 8.7779 | 1.70E-10 | *Sparc* | -3.5875 | 1.71E-09 |
| *H2-M2* | 8.7014 | 3.92E-10 | *Fcer2a* | -3.339 | 2.41E-05 |
| *Edn1* | 8.6207 | 9.30E-10 | *Gabbr1* | -3.2659 | 2.02E-12 |

**Table S3. List of the top 20 significantly upregulated and downregulated DEGs comparing lung AMs from mice infected with *C. neoformans* at 7 dpi to those treated with PBS.**
