# Supplementary material for: Transcriptomic profiling of lung alveolar macrophages reveals distinct contribution of sterol metabolism in macrophage response to Cryptococcus gattii infection
Source: PLoS One. 2025 Sep 30;20(9):e0333090. doi: 10.1371/journal.pone.0333090 (PMC12483273; doi:10.1371/journal.pone.0333090)
Supplement: S4 Table — (DOCX) [file pone.0333090.s004.docx]

| Upregulated | | | Downregulated | | |
| --- | --- | --- | --- | --- | --- |
| Gene | Log_2_ Fold Change | P-value | Gene | Log_2_ Fold Change | P-value |
| *Hcn3* | 4.9558 | 1.93E-09 | *Arhgef39* | -2.6012 | 7.47E-06 |
| *Ereg* | 4.778 | 5.25E-06 | *Plin1* | -2.3066 | 1.74E-05 |
| *Abca8a* | 4.2129 | 2.15E-06 | *Abca1* | -2.2013 | 3.99E-07 |
| *Aebp1* | 3.9287 | 8.90E-08 |  |  |  |
| *Fkbp11* | 3.8431 | 6.18E-13 |  |  |  |
| *Cd200r2* | 3.7435 | 6.77E-12 |  |  |  |
| *Pamr1* | 3.4074 | 2.23E-07 |  |  |  |
| *Il6* | 3.1936 | 1.44E-05 |  |  |  |
| *Syngr1* | 3.1506 | 1.55E-09 |  |  |  |
| *Gng8* | 3.1491 | 7.20E-06 |  |  |  |
| *Nr4a3* | 2.8538 | 1.65E-09 |  |  |  |
| *Fdps* | 2.8203 | 1.82E-10 |  |  |  |
| *Lif* | 2.7755 | 9.37E-09 |  |  |  |
| *Il1b* | 2.7573 | 3.24E-10 |  |  |  |
| *Idi1* | 2.7417 | 1.08E-09 |  |  |  |
| *Nr4a2* | 2.6923 | 1.50E-08 |  |  |  |
| *Car4* | 2.6587 | 1.10E-09 |  |  |  |
| *Cyp51* | 2.6493 | 1.66E-09 |  |  |  |
| *Sqle* | 2.6096 | 3.03E-09 |  |  |  |
| *Acsl3* | 2.5769 | 1.92E-08 |  |  |  |

**Table S4. List of the top 20 significantly upregulated and 3 significantly downregulated DEGs comparing lung AMs from mice infected with *C. gattii* at 7 dpi to those treated with PBS.**
