# Supplementary material for: Transcriptomic profiling of lung alveolar macrophages reveals distinct contribution of sterol metabolism in macrophage response to Cryptococcus gattii infection
Source: PLoS One. 2025 Sep 30;20(9):e0333090. doi: 10.1371/journal.pone.0333090 (PMC12483273; doi:10.1371/journal.pone.0333090)
Supplement: S5 Table — (DOCX) [file pone.0333090.s005.docx]

| GO:ID | Pathway | P-value | Upregulated | |
| --- | --- | --- | --- | --- |
|  |  |  | Number | Gene name |
| GO:0016126 | sterol biosynthetic process | 3.13E-11 | 11 | Cyp51/Fdft1/Hmgcr/Hsd17b7/Msmo1/Sc5d/Lss/Dhcr24/Ch25h/Fdps/Hmgcs1 |
| GO:0016125 | sterol metabolic process | 1.67E-09 | 13 | Cyp51/Fdft1/Hmgcr/Sqle/Hsd17b7/Msmo1/Sc5d/Ldlr/Lss/Dhcr24/Ch25h/Fdps/Hmgcs1 |
| GO:1901617 | organic hydroxy compound biosynthetic process | 2.33E-09 | 14 | Cyp51/Fdft1/Hmgcr/Epas1/Hsd17b7/Nr4a2/Il1b/Msmo1/Sc5d/Lss/Dhcr24/Ch25h/Fdps/Hmgcs1 |
| GO:0006694 | steroid biosynthetic process | 3.35E-09 | 13 | Cyp51/Igf1/Fdft1/Hmgcr/Hsd17b7/Il1b/Msmo1/Sc5d/Lss/Dhcr24/Ch25h/Fdps/Hmgcs1 |
| GO:0006695 | cholesterol biosynthetic process | 3.35E-09 | 9 | Cyp51/Fdft1/Hmgcr/Hsd17b7/Sc5d/Lss/Dhcr24/Fdps/Hmgcs1 |
| GO:1902653 | secondary alcohol biosynthetic process | 3.51E-09 | 9 | Cyp51/Fdft1/Hmgcr/Hsd17b7/Sc5d/Lss/Dhcr24/Fdps/Hmgcs1 |
| GO:0008202 | steroid metabolic process | 4.04E-09 | 16 | Cyp51/Igf1/Fdft1/Hmgcr/Sqle/Hsd17b7/Dhrs9/Il1b/Msmo1/Sc5d/Ldlr/Lss/Dhcr24/Ch25h/Fdps/Hmgcs1 |
| GO:0008203 | cholesterol metabolic process | 4.04E-09 | 12 | Cyp51/Fdft1/Hmgcr/Sqle/Hsd17b7/Sc5d/Ldlr/Lss/Dhcr24/Ch25h/Fdps/Hmgcs1 |
| GO:1902652 | secondary alcohol metabolic process | 5.42E-09 | 12 | Cyp51/Fdft1/Hmgcr/Sqle/Hsd17b7/Sc5d/Ldlr/Lss/Dhcr24/Ch25h/Fdps/Hmgcs1 |
| GO:0044283 | small molecule biosynthetic process | 1.11E-08 | 19 | Cyp51/Fads1/Fdft1/Hmgcr/Fads2/Scd2/Il6/Hsd17b7/Dhrs9/Il1b/Acss2/Msmo1/Sc5d/Acsl3/Lss/Dhcr24/Ch25h/Fdps/Hmgcs1 |
| GO:0008610 | lipid biosynthetic process | 2.29E-08 | 20 | Cyp51/Fads1/Igf1/Fdft1/Hmgcr/Fads2/Scd2/Hsd17b7/Dhrs9/Il1b/Acss2/Msmo1/Sc5d/Ldlr/Acsl3/Lss/Dhcr24/Ch25h/Fdps/Hmgcs1 |
| GO:0046165 | alcohol biosynthetic process | 1.18E-07 | 10 | Cyp51/Fdft1/Hmgcr/Hsd17b7/Il1b/Sc5d/Lss/Dhcr24/Fdps/Hmgcs1 |
| GO:1901615 | organic hydroxy compound metabolic process | 3.48E-07 | 16 | Cyp51/Fdft1/Hmgcr/Sqle/Epas1/Hsd17b7/Nr4a2/Il1b/Msmo1/Sc5d/Ldlr/Lss/Dhcr24/Ch25h/Fdps/Hmgcs1 |
| GO:0030595 | leukocyte chemotaxis | 3.48E-07 | 12 | Tnfsf14/Csf1/Cxcl16/Ccl9/Adam8/Il1b/Spp1/Itgam/Ccl22/Ccl17/Ch25h/Slamf8 |
| GO:0006066 | alcohol metabolic process | 8.74E-07 | 13 | Cyp51/Fdft1/Hmgcr/Sqle/Hsd17b7/Il1b/Sc5d/Ldlr/Lss/Dhcr24/Ch25h/Fdps/Hmgcs1 |

**Table S5. List of the significant GO enrichment analyses of upregulated DEGs comparing lung AMs from mice infected with *C. gattii* to those treated with PBS.**
