# Supplementary material for: Transcriptomic profiling of lung alveolar macrophages reveals distinct contribution of sterol metabolism in macrophage response to Cryptococcus gattii infection
Source: PLoS One. 2025 Sep 30;20(9):e0333090. doi: 10.1371/journal.pone.0333090 (PMC12483273; doi:10.1371/journal.pone.0333090)
Supplement: S6 Table — (DOCX) [file pone.0333090.s006.docx]

| GO:ID | Pathway | P-value | Downregulated | |
| --- | --- | --- | --- | --- |
|  |  |  | Number | Gene name |
| GO:0097006 | regulation of plasma lipoprotein particle levels | 0.005591 | 3 | Abca1/Pltp/Mylip |
| GO:0010875 | positive regulation of cholesterol efflux | 0.010541 | 2 | Abca1/Pltp |
| GO:0032373 | positive regulation of sterol transport | 0.010541 | 2 | Abca1/Pltp |
| GO:0032376 | positive regulation of cholesterol transport | 0.010541 | 2 | Abca1/Pltp |
| GO:0010874 | regulation of cholesterol efflux | 0.010541 | 2 | Abca1/Pltp |
| GO:0032371 | regulation of sterol transport | 0.019256 | 2 | Abca1/Pltp |
| GO:0032374 | regulation of cholesterol transport | 0.019256 | 2 | Abca1/Pltp |
| GO:0071827 | plasma lipoprotein particle organization | 0.019256 | 2 | Abca1/Pltp |
| GO:0071825 | protein-lipid complex subunit organization | 0.019256 | 2 | Abca1/Pltp |
| GO:0033344 | cholesterol efflux | 0.021412 | 2 | Abca1/Pltp |
| GO:0016323 | basolateral plasma membrane | 0.027195 | 3 | Cd300lg/Atp2b4/C5ar1 |
| GO:0005811 | lipid particle | 0.027195 | 2 | Cidec/Plin1 |
| GO:0045428 | regulation of nitric oxide biosynthetic process | 0.031366 | 2 | Atp2b4/Tlr5 |
| GO:0032370 | positive regulation of lipid transport | 0.031366 | 2 | Abca1/Pltp |
| GO:0030301 | cholesterol transport | 0.031366 | 2 | Abca1/Pltp |

**Table S6. List of the most significant GO enrichment analyses of downregulated DEGs comparing lung AMs from mice infected with *C. gattii* to those treated with PBS.**
