# Supplementary material for: Transcriptomic profiling of lung alveolar macrophages reveals distinct contribution of sterol metabolism in macrophage response to Cryptococcus gattii infection
Source: PLoS One. 2025 Sep 30;20(9):e0333090. doi: 10.1371/journal.pone.0333090 (PMC12483273; doi:10.1371/journal.pone.0333090)
Supplement: S1 Fig — (DOCX) [file pone.0333090.s007.docx]

**
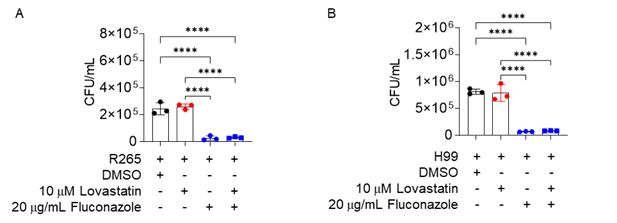
**

**Fig.** **S1. Direct effects of lovastatin treatment on the in vitro growth of *C. gattii* and *C. neoformans*.**

*C gattii* R265 and *C. neoformans* H99 were cultured at a concentration of 1 x 10^5^ cells/mL in serum-free DMEM and treated with either vehicle control (DMSO), lovastatin (10 μM), fluconazole (20 μg/mL), or a combination of fluconazole (20 μg/mL) and lovastatin (10 μM) for 24 hours at 37°C. After treatment, CFUs were assessed. Representative CFU data for (A) R265 and (B) H99 were presented in CFU/mL. Graphs depict mean ± SD of three independent experiments. Significance was determined using one-way ANOVA followed by Turkey post hoc analysis (*****p*<0.0001).
