# Supplementary material for: Transcriptomic profiling of lung alveolar macrophages reveals distinct contribution of sterol metabolism in macrophage response to Cryptococcus gattii infection
Source: PLoS One. 2025 Sep 30;20(9):e0333090. doi: 10.1371/journal.pone.0333090 (PMC12483273; doi:10.1371/journal.pone.0333090)
Supplement: S2 Fig — (DOCX) [file pone.0333090.s008.docx]

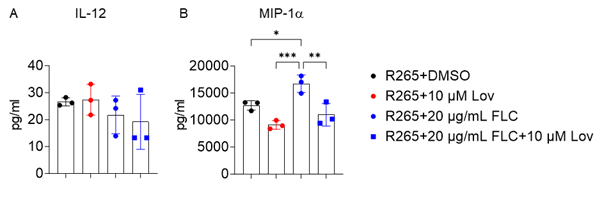


**Fig. S2. IL-12 and MIP-1α secretion in *C. gattii*-infected BMDM culture supernatants.**

Culture supernatants from BMDMs under various conditions were analyzed for (A) IL-12 and (B) MIP-1α secretion using the Bio-Plex Mouse Cytokine Assays (Biorad Laboratories). Graphs depict mean ± SD of three independent experiments. Significance was determined using one-way ANOVA followed by Turkey post hoc analysis (**p*<0.05, ***p*<0.01, ****p*<0.001). Experimental conditions: R265+DMSO: R265-infected cells treated with DMSO, R265+10 µM Lov, R265-infected cells treated with 10 µM lovastatin, R265+20 µg/ml FLC, R265-infected cells treated with 20 µg/ml fluconazole, R265+20 µg/mL FLC+10 µM Lov, R265-infected cells treated with fluconazole (20 µg/mL) and lovastatin (10 µM).
