# Supplementary material for: Transcriptomic profiling of lung alveolar macrophages reveals distinct contribution of sterol metabolism in macrophage response to Cryptococcus gattii infection
Source: PLoS One. 2025 Sep 30;20(9):e0333090. doi: 10.1371/journal.pone.0333090 (PMC12483273; doi:10.1371/journal.pone.0333090)
Supplement: S3 Fig — (DOCX) [file pone.0333090.s009.docx]

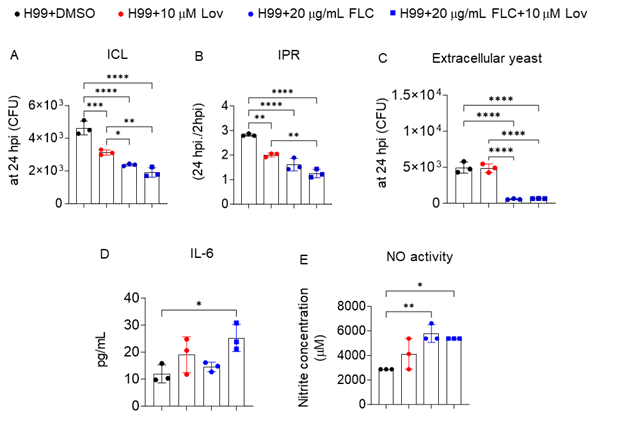


**Fig. S3. Effect of lovastatin and its combination with fluconazole on *C. neoformans*-infected BMDMs.**

BMDMs were infected with *C. neoformans* H99. Two hours post-infection, non-phagocytosed yeast cells were removed, and macrophages were subsequently treated with DMSO, lovastatin (10 µM), fluconazole (20 µg/mL), or combination of fluconazole (20 µg/mL) and lovastatin (10 µM) followed by the evaluation of ICL, IPR, and extracellular yeast at 24 hpi by the CFU assay. (A) The intracellular cryptococcal load (ICL) at 24 hpi in BMDMs. Data are presented as CFU. (B) The intracellular proliferation rate (IPR) in BMDMs. Data are presented as the ratio of CFU of intracellular cryptococci at 24 hpi to the CFU measured at 2 hpi. (C) The extracellular yeasts using the CFU assay at 24 hpi in BMDMs. Data are presented as CFU. (D) ELISA analysis of IL-6 secretion in culture supernatant of BMDMs treated with indicated treatment conditions. (E) Nitric Oxide (NO) activity was detected in culture supernatant of BMDMs treated with indicated treatment conditions. The y-axis indicated the concentration of nitrite (μM). Graphs depict mean ± SD of three independent experiments. Significance was determined using one-way ANOVA followed by Turkey post hoc analysis (**p*<0.05, ***p*<0.01, ****p*<0.001, *****p*<0.0001). Experimental conditions: H99+DMSO: H99-infected cells treated with DMSO, H99+10 µM Lov, H99-infected cells treated with 10 µM lovastatin, H99+20 µg/ml FLC, H99-infected cells treated with 20 µg/ml fluconazole, H99+20 µg/mL FLC+10 µM Lov, H99-infected cells treated with fluconazole (20 µg/mL) and lovastatin (10 µM).
